# Supplementary material for: In vivo vizualisation of mono-ADP-ribosylation by dPARP16 upon amino-acid starvation
Source: eLife. 2016 Nov 22;5:e21475. doi: 10.7554/eLife.21475 (PMC5127640; doi:10.7554/eLife.21475)
Supplement: Figure 1—source data 1. — DOI: http://dx.doi.org/10.7554/eLife.21475.003 [file elife-21475-fig1-data1.docx]

*Figure 1.Supplement figure 1*. Aguilera Gomez et al, 2016

| **Primers used for cloning and RNAi** | |
| --- | --- |
| sfGFP-F | ggccgcggatggtgagcaagggcgagga |
| sfGFP-R | gggtttaaacttacttgtacagctcgtccatg |
| dARTD1/PARP1-F | cagtggtaccatggatattgaattaccttatcttgctgagtatgc |
| dARTD1/PARP1-R | cagtgggccctcataagaatacttgaattccatacgaaacaaatattgaatatt |
| ARTD5-6/Tankyrase-F | gtcaggtaccatggccaacagcagccgaagtcg |
| ARTD5-6/Tankyrase-R | gtcagggccctctcttgtatcctccgttcc |
| ARTD15/PARP16-F | gtcaggtaccatgactctgctttcgggcgccaac |
| ARTD15/PARP16-R | gtcagggccctccaaaatacctcgcttgaatacgtagaatacgtagaagcc |
| pMT-V5-PARP16-F | gtcaggtaccatgactctgctttcgggcgccaac |
| pMT-V5-PARP16-R | gaatccgttcgcctgggcttcgctggcygcaagctagagaagg |
| pMT-Y199>A-PARP16-sfGFP | ccttctctagcttgcagccagcgaagcccaggcgaacggattc |
| pMT-V5-Y221>A-PARP16-F | caaaattccctggacaatggaaaggctctggagctaacctgcgatatc |
| pMT-V5-Y221>A-PARP16-R | gatatcgcaggttagctccagagcctttccattgtccagggaattttg |
| pMT-V5-ΔTM-PARP16 | gtcagggccctcggatatggccttcttgtggtatctttcc |
| dARTD1/PARP1-RNAi-F | ctaatacgactcactatagggcgaaggaaattaacggatcttgtgga |
| dARTD1/PARP1-RNAi-R | ctaatacgactcactatagggcgaagattttattggtggtatggcg |
| ARTD5-6/Tankyrase-RNAi-F | taatacgactcactatagggcgagctgaggtccaccacaggcattg |
| ARTD5-6/Tankyrase-RNAi-R | taatacgactcactatagggcgatcttgtatcctccgttccactactgc |
| ARTD15/PARP16-RNAi-F | taatacgactcactatagggcgaatgactctgctttcgggcgccaac |
| ARTD15/PARP16-RNAi-R | taatacgactcactatagggcgacaaaatacctcgcttgaatacgtagaagcc |
| ARTD15/PARP16-RNAi-3utr-F | taatacgactcactatagggcgacttttactatccatatcctgtgacc |
| ARTD15/PARP16-RNAi-3utr-R | taatacgactcactatagggcgattcacacattacagatgcct |
| ARDT8-Macro1-3-F | cagtaccggtgggcagaagtgcttctctcggac |
| ARDT8-Macro1-3-R | cagtgtttaaaccccttctcttttcttcatgttggcataaaacac |
| Hex-HIS-TEV-F | 5Phos/ccggtcatcatcaccatcaccatgagaacctgtacttccaatccata |
| Hex-HIS-TEV-R | 5Phos/ccggtattggattggaagtacaggttctcatggtgatggtgatgatga |
| GFP-MAD-MutMacro2-F | ctaagtccctcttggaaaaagctgaaccagagctccaggaggaattg |
| GFP-MAD-MutMacro2-R | caattcctcctggagctctggttcagctttttccaagagggacttag |
| YFP-F | gactgggcccgcggttcgaaatggtgtctaaaggggaagagctcttta |
| YFP-R | gactaccggtcttatacagctcatccatgcccaggg |
| H2A1.1-F | cagtaccggtcagggtgaagtcagtaaggcagcc |
| H2A1.1-R | cagtgtttaaacgttggcgtccagcttggccatttc |
| CAAX-RAS-F | cagtaccggttccggactcagatctcgagctc |
| CAAX-RAS-R | cagtgtttaaacttacataattacacactttgtctttgacttctttttc |
| SEC16>NC1-F | cagtactagtatgcattcaaccactcagcaggagaagaa |
| SEC16-R | ccaccggtaatggggctgccatacgtttgc |
| SEC16>CT-R | gtcaccgcggaaatgagacacgattaatgagaatgggatcatg |
| SEC16-CT-F | cagtggtaccatggcgagtcctccgaatgccactag |
| SEC16-SRD-F | cagtggtaccatgctgcagcaacagacgcgccc |
| SEC16-SRD-R | gtcaccgcggaatggggctgccatacgtttgcg |
| SEC16-SRD-C-F | cagtggtaccatgaataaaaatgcgggctggtttggcg |
| SEC16-SRD-C-R | gtcaccgcggggtgtttgtccagcatttgcgctc |
| SEC16 ΔSRD-C-fusion-F | cgcacctggggaaggcaacggcgacgaggctgaaag |
| SEC16 ΔSRD-C-fusion-R | cgttgccttccccaggtgcgccaagatcgctgcc |
| SEC16 ΔSRD-C-F | gcgcccggcgatatccatgcccaag |
| SEC16 ΔSRD-C-R | gtcaccgcggctgctggggtacaaagtaaccgc |
